# Stroke and Novel Coronavirus Infection in Humans: A Systematic Review and Meta-Analysis

**DOI:** 10.3389/fneur.2020.579070

**Published:** 2020-10-06

**Authors:** Kai Wei Lee, Abdul Hanif Khan Yusof Khan, Siew Mooi Ching, Peck Kee Chia, Wei Chao Loh, Anna Misya'il Abdul Rashid, Janudin Baharin, Liyana Najwa Inche Mat, Wan Aliaa Wan Sulaiman, Navin Kumar Devaraj, Dhashani Sivaratnam, Hamidon Basri, Fan Kee Hoo

**Affiliations:** ^1^Department of Family Medicine, Faculty of Medicine and Health Sciences, Universiti Putra Malaysia, Serdang, Malaysia; ^2^Department of Medicine, Faculty of Medicine and Health Sciences, Universiti Putra Malaysia, Serdang, Malaysia; ^3^Malaysian Research Institute on Ageing, Universiti Putra Malaysia, Serdang, Malaysia; ^4^Department of Ophthalmology, Faculty of Medicine and Health Sciences, Universiti Putra Malaysia, Serdang, Malaysia

**Keywords:** stroke, cerebrovascular disease, COVID-19, coronavirus (2019-nCoV), systematic review, meta-analysis, SARS-CoV-2

## Abstract

**Background:** As the world witnessed the devastation caused by the coronavirus disease 2019 (COVID-19) outbreak, a growing body of literature on COVID-19 is also becoming increasingly available. Stroke has increasingly been reported as a complication of COVID-19 infection. However, a systematic synthesis of the available data has not been conducted. Therefore, we performed a systematic review and meta-analysis of currently available epidemiological, clinical, and laboratory data related to both stroke and COVID-19 infection.

**Methods:** We systematically searched Medline, Cinahl, and PubMed for studies related to stroke and COVID-19 from inception up to June 4, 2020. We selected cohort studies, case series, and case reports that reported the occurrence of stroke in COVID-19 patients. A fixed-effects model was used to estimate the pooled frequency of stroke in COVID-19 patients with a 95% confidence interval (CI).

**Results:** Twenty-eight studies were included in the systematic review and seven studies for the meta-analysis. The pooled frequency of stroke in COVID-19 patients was 1.1% (95% CI: 0.8, 1.3). The heterogeneity was low (*I*^2^ = 0.0%). Even though the frequency of stroke among patients having COVID-19 infection was low, those with concomitant COVID-19 infection and stroke suffered from a more severe infection and eventually had a poorer prognosis with a higher mortality rate (46.7%) than COVID-19 alone. Many COVID-19 patients shared the common traditional risk factors for stroke. We noted that ischemic stroke involving the anterior circulation with large vessels occlusion is the most common type of stroke with more strokes seen in multi-territorial regions, suggesting systemic thromboembolism. An elevated level of D-dimers, C-reactive protein, ferritin, lactic acid dehydrogenase, troponin, ESR, fibrinogen, and a positive antiphospholipid antibody were also noted in this review.

**Conclusions:** The occurrence of stroke in patients with COVID-19 infection is uncommon, but it may pose as an important prognostic marker and indicator of severity of infection, by causing large vessels occlusion and exhibiting a thrombo-inflammatory vascular picture. Physicians should be made aware and remain vigilant on the possible two-way relationship between stroke and COVID-19 infection. The rate of stroke among patients with COVID-19 infection may increase in the future as they share the common risk factors.

## Introduction

In December 2019, an outbreak of a novel respiratory infection was first detected in Wuhan, China, linked to three cases of patients presenting with pneumonia (1, 2). The cause of the pneumonia was found to be a viral infection known as novel coronavirus disease (COVID-19), and by March 2020, the World Health Organization (WHO) declared this disease as a pandemic caused by a virus known as SARS-CoV-2 (severe acute respiratory syndrome coronavirus 2) (3, 4). The WHO stated in its report on the state of the world's health that humans are now facing a serious threat from COVID-19 (4, 5), and it was now necessary to declare COVID-19 as a public health emergency (6).

COVID-19's main presentation relates to the infection of the upper respiratory system, with clinical features such as fever, dry cough, myalgia, and malaise, and in more severe cases, patients may develop pneumonia that may proceed to the life-threatening acute respiratory distress syndrome (ARDS) (7). Patients infected with COVID-19 will also experience several mild neurological symptoms such as headache, dizziness and anosmia, to severe symptoms like altered level of consciousness, acute cerebrovascular events, seizures, and ataxia (8, 9). In addition, COVID-19 could also cause viral encephalitis and hemorrhagic necrosis in the mesial temporal lobes and thalamus (10–12). Stroke is one of the more disabling neurological complications being reported, where the first retrospective cohort of COVID-19 showed stroke occurrence in around 2% of the patients (13). The American Stroke Association indicated that the risk of stroke doubled every 10 years after the age of 55, and therefore, stroke affects more older adults than younger ones (14, 15). However, due to COVID-19, literature has reported an increasing number of premature strokes in the younger generation (16).

The pathophysiology for the development of stroke in patients with COVID-19 is multifactorial. Infection, in general, may increase the odds of stroke 1.4-fold, particularly in the early convalescence phase, and this association may also be similarly expected among COVID-19 patients (17). Secondly, SARS-CoV-2 may potentially predispose to thrombogenesis and increase the risk of stroke by infecting the myocardium cells via ACE2 (angiotensin-converting enzyme II) receptor and causing vascular injury and inflammation (18). COVID-19 has been shown to create a prothrombotic state as evidenced by high D-dimer titres that further propagate the risk of thrombosis (19). Moreover, COVID-19 patients appear to be in a hyper inflammation state or cytokine storm like condition, which resulted in secretion of high interleukin-6 (IL-6) levels, which in turn translates to hyperviscosity and increases the risk for stroke propensity (20). Apart from the increased thrombotic potential in large vessels in patients with COVID-19, the patient may also be susceptible to spontaneous intracerebral hemorrhage and micro thrombosis of small penetrating vessels owing to the potential risk of vascular endothelial damage (21). There is growing evidence of the development of thromboembolic complications among patients with COVID-19, the occurrence of stroke. Several case studies have also shown that patients with pre-existing cerebrovascular disease may be at a higher risk for a poor outcome if they become infected with COVID-19 (22–24). Given the worldwide COVID-19 cases are now over nine million as updated on June 26, 2020, and still rising in an exponential manner (25), the understanding of the association between stroke and COVID-19 is essential in order to prevent debilitating sequelae associated with stroke and to aid in the prevention and management in these groups of patients.

### Significance of the Study

Due to the novelty of the virus and the relatively short duration of the current COVID-19 outbreak, only a limited and scattered body of scientific evidence on the neurological complications of COVID-19 is currently available. Furthermore, the possible two-way association between COVID-19 and stroke has not yet been elucidated, and currently there are only limited data available on stroke co-occurrence and characterization in patients with COVID-19, which urgently needs further investigation and analysis to ensure a better outcome for this group of patients. Therefore, it is vital to perform this review in order to determine the frequency of stroke among COVID-19 patients and stroke characterization, as this may impact future management.

We, therefore, performed a systematic review and meta-analysis involving the epidemiological, clinical presentation, imaging characteristics, and laboratory finding related to both stroke and COVID-19 infection.

## Methods

This systematic review study was registered with the Medical Research and Ethics Committee, Ministry of Health Malaysia (registration number: NMRR-20-1200-55395) and was conducted according to Preferred Reporting Items for Systematic Reviews and Meta-Analyses (PRISMA) (26) (Appendix 1).

### Literature Search

Two investigators (AHKYK and JB) independently searched the Medline, Cinahl, and PubMed databases for potential studies that were published in peer-reviewed journals from inception to June 4, 2020. We used the following search terms: (Cerebrovascular Accident OR CVA OR Stroke) AND (COVID-19 OR CORONAVIRUS OR 2019-NCOV) with limiters of ENGLISH and HUMAN. The search strategies with the Boolean or phrase operators are shown in Appendix 2. Subsequently, we removed duplications using Endnote® before the next process of screening the title and abstracts for suitability. Finally, the selected articles with their full text were assessed for their eligibility to be recruited into this systematic review and meta-analysis.

### Study Selection

All relevant articles identified through the above comprehensive databases were imported into the Endnote® programme version X5. Initially, we performed de-duplication. Title and abstracts were then reviewed for their relevance and articles highlighting cases of COVID-19 and its relevance to stroke were reviewed in full text by four investigators (AHKYK, JB, PKC, and WCL) who are clinical neurologists with not <5 years of experience in the field of clinical neurology. Studies were selected based on inclusion criteria that these studies have data on the frequency of stroke in cases of COVID-19 or possess any data relevant to the relative risk of COVID-19 and stroke. Studies were excluded if they are a review paper, or there is no required data for both these conditions. We also excluded any study with patients who developed stroke prior to COVID-19 infection. Any disagreements between the investigators were resolved through discussions and consultations with another two senior investigators (SMC and FKH) before the final consensus for quantitative analysis was reached.

### PICO (Participants/Population, Intervention/Exposure, Comparator/Control, Outcomes)

The participants should be those (age >18 years) with or without a confirmed diagnosis of stroke. Exposure was referred to as exposure to COVID-19 disease, whereby there were no limitations in severity criteria. Comparator was referred to as non-COVID-19 disease and COVID-19 patients without stroke. The main outcomes we examined in this review were percentage or frequency of stroke that occurs after COVID-19 infection, whereby the stroke incidence could be an ischemic and hemorrhagic stroke, venous stroke due to venous sinus thrombosis, or transient ischemic attack. The secondary outcomes were clinical presentation, the subtype of stroke, imaging characteristics, and laboratory finding related to both stroke and COVID-19 infection.

### Data Extraction

Four investigators were paired into two groups (group 1: AHKYK and JB; group 2: PKC and WCL) to perform the data extraction independently. The following data were extracted from every study: the last name of the first author, year of publication, country, severity status, study design, patient characteristics (ethnicity composition, gender, and mean age), comorbidities (diabetes, hyperlipidemia, hypertension, ischemic heart disease, heart failure, previous stroke, chronic kidney disease/end-stage renal disease, number of stroke patients per overall participants, any information relevant to strokes such as the location of stroke [arterial or venous]), types of stroke (ischemic or haemorrhagic), classification of stroke, mortality rate, and blood parameters. Another two investigators (AMAR and LNIM) performed proofreading to ensure no errors and bias in the data extraction.

### Strategy for Data Synthesis

Pooled frequency of stroke among COVID-19 patients was estimated using meta-analysis, and the data required for this was the number of patients with stroke and COVID-19 infection (nominator) divided by the total number of patients with COVID-19 infection (denominator). A synthesis of the findings in the aspect of clinical presentation, imaging characteristics, and laboratory finding extracted from included studies were summarized in tables. Pertaining to clinical presentation, we classified stroke based on vessels occlusion and TOAST, whereby data were presented either in N value or ultimate decision-maker (Yes/No). The ultimate decision, either Yes or No, was used because the particular study had only one patient with stroke. Classification of stroke was based on imaging finding such as arterial vs. venous; ischemic vs. hemorrhagic; location of stroke (anterior circulation, posterior circulation, or multiple territories), whereby data were presented either in *N* value or ultimate decision-maker (Yes/No). Laboratory findings with clinical importance to inflammation due to stroke or viral infection were also examined, which include erythrocyte sedimentation rate, C-reactive protein, ferritin, D-dimer, lactic acid dehydrogenase, fibrinogen, antiphospholipid, procalcitonin, interleukin6, troponin, platelet, and prothrombin time. Blood parameters were presented in mean ± standard deviation or range.

### Quality Assessment

The quality of the individual studies pertaining to cohort studies was determined using the checklist Strengthening the Reporting of Observational Studies in Epidemiology (STROBE), which has 22 items that assess components in observational studies (27). A “0” was given if that item was not reported; “1” was awarded if that item was sufficiently shown in the article. Each article's quality was graded as “good” if STROBE scores ≥14/22 or graded as “poor” if strobe score <14/22 (27). Nevertheless, studies would have been included in this review regardless of the STROBE grading.

We used a quality appraisal checklist for case series studies developed by the Institute of Health Economics, which appraises over 20 items. This is a three-options checklist with Yes/Partial/Unclear/No depending on the clarity of items presented in case series (28) (Appendix 3).

### Statistical Analysis

A fixed-effect (DerSimonian and Laird method) meta-analysis method was employed to calculate the pooled frequency from these related studies, and it was reported with a 95% confidence interval (CI). I^2^ index was used to assess the study's heterogeneity (i.e., low is <25%, moderate 25–50%, and high >50%) that indicated the total percent of discrepancy due to variation in the included studies (29). We also examined publication bias by Begg's test and Egger's test for studies which entered meta-analysis (30). A sensitivity analysis was conducted using leave-one-out meta-analysis to examine how individual studies affect the overall estimation of the rest of the studies. For statistical analysis, Open Meta(Analyst)® software was used, and this software can be accessed and downloaded from http://www.cebm.brown.edu/openmeta/index.html (31).

## Results

### Description of Included Studies

**Figure 1:**
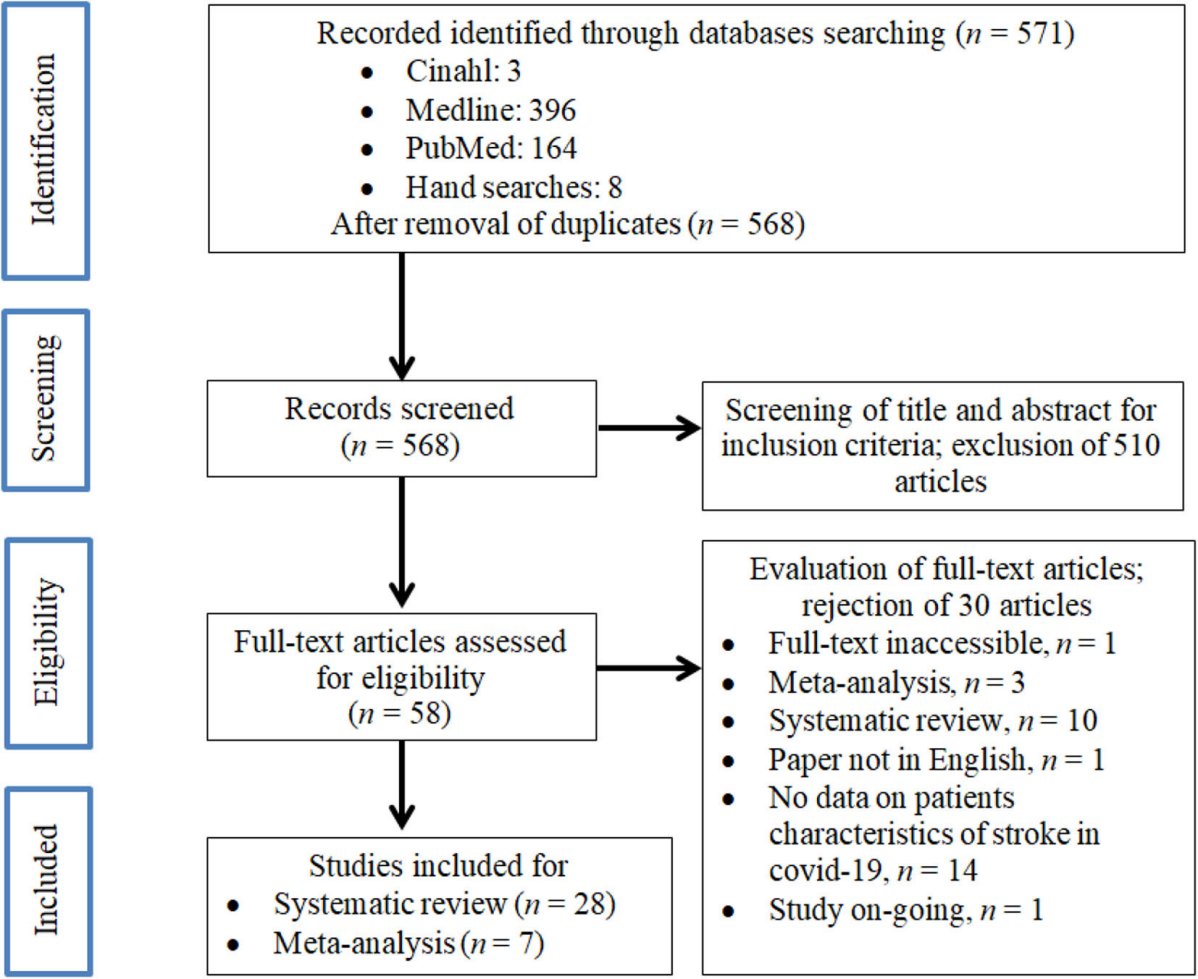
PRISMA flow diagram of the literature screening process.

We identified 571 manuscripts in the initial screening, as shown in Figure 1. After removal of duplicate articles (*n* = 3), a total of 568 studies were retrieved for further assessment. After screening for its suitability through the individual title and abstract, 58 studies fulfilled both our inclusion and exclusion criteria. After careful evaluation, 28 articles were finally included for the systematic review and seven studies for the meta-analysis.

#### Characteristics of Included Studies

The main characteristics of the included studies are shown in Table 1. A total sample of 8,771 participants was included in the systematic review. These studies were conducted in many countries worldwide including in China (13, 40, 43), France (37, 39, 48, 50), India (42), Iran (51), Italy (32, 33, 46), the Netherlands (34), Philippines (52), Spain (44, 53), Turkey (58), UK (41, 55), and the USA (21, 35, 36, 45, 47, 49, 54, 56, 57). Out of 27 studies, eight studies were of retrospective cohort study design, 11 were case series, and nine were case reports. The mean age of the participants ranged from 36 to 81 years old, giving a grand mean age of participants from the included studies of 62.9 ± 12.2 years, with more than half of them being males (64.1%). The overall mortality rate among stroke patients ranged from 22.2 to 43.0%; the average mortality rate for stroke patients with COVID-19 and non-COVID-19 infection were 46.7 and 8.7%, respectively. A majority of the respondents were diagnosed with COVID-19 using the reverse transcriptase-polymerase chain reaction (RT-PCR) tests conducted on samples collected either from the nasopharyngeal or oropharyngeal swab, and some also had concurrent confirmation by the antibody serology test.

**Table 1:** Characteristics of the included studies.

**References**	**Year**	**Country**	**Study design**	**Quality assessment**	**Study period**	**Patient characteristics (%)**		**Frequency of stroke**		**Number of mortality among stroke patients (rate, %)**	**Number of mortality among stroke patients with COVID+ (%)**	**Number of mortality among non-COVID stroke patients (%)**
						**Gender, %**	**Age was presented either in mean ± standard deviation or median (Interquartile range)**	**No. patients with stroke**	**Total no. of participants**			
Lodgiani et al.* (32)	2020	Italy	RC	Good	13 Feb−10 April	Male (68); Female (32)	66 (range 5–75)	9	388	2 (22.2)	Unclear	Unclear
Benussi et al. (33)	2020	Italy	RC	Good	21 Feb−5 April	Male (51.2); Female (48.8)	76.9 (range 66.8–85.2)	43	56	19 (17.1)	15 (34.9)	4 (5.9)
Klok et al.* (34)	2020	Netherland	RC	Poor	7 Mar−15 April	Male (76); Female (24)	64 ± 15.5	3	184	N/A	N/A	N/A
Jain et al.* (35)	2020	USA	RC	Good	1 Mar−13 April	Male (60.7); Female (39.3)	64 (range 2 weeks−105)	35	3218	15 (43.0)	Unclear	Unclear
Yaghi et al. (36)	2020	USA	RC	Good	15 April−19 April	Male (71.9); Female (28.1)	63 (IQR 17)	32	3556	18 (27.7)	14 (63.6)	4 (9.3)
Escalard et al. (37)	2020	France	RC	Poor	1 Mar−15 April	Male (80); Female (20)	59.5 (IQR54–71.5)	10	37	9 (24.3)	6 (38)	3 (11)
Helms et al.* (39)	2020	France	PC	Good	4 Mar−31 Mar	Male (81.3); Female (18.7)	63 (1QR 53–71)	2	150	N/A	N/A	N/A
Xiong et al.* (40)	2020	China	RC	Good	18 Jan−20 Mar	Male (55); Female (45)	48.7 ± 17.1	10	917	3 (33.3)	N/A	N/A
Mao et al.* (13)	2020	China	CS	Appendix 3	16 Jan−19 Feb	Male (40.7); Female (59.3)	52.7 ± 15.5	6	214	N/A	N/A	N/A
Beyrouti et al. (41)	2020	UK	CS	Appendix 3	1 April−16 April	Male (83.3); Female (16.7)	69.8 ± 12.7	6	N/A	N/A	1 (16.6)	N/A
Avula et al. (42)	2020	India	CS	Appendix 3	N/A	Male (25); Female (75)	81 ± 5.4	4	N/A	N/A	3 (75.0)	N/A
Zhang et al. (43)	2020	China	CS	Appendix 3	N/A	Male (66.7); Female (33.3)	68 ± 2.7	3	N/A	N/A	N/A	N/A
Barios Lopez et al. (44)	2020	Spain	CS	Appendix 3	25 Mar−17 April	Male (50); Female (50)	71.5 ± 15.3	4	N/A	N/A	2 (50.0)	N/A
Oxley et al. (45)	2020	USA	CS	Appendix 3	23 Mar−7 April	Male (80); Female (20)	40.4 ± 6.2	5	N/A	N/A	N/A	N/A
Tunc et al. (Tunç et al., 2020)	2020	Turkey	CS	Appendix 3	1 April−14 April	Male (50); Female (50)	65.3 ± 12.2	4	N/A	N/A	0 (0.0)	N/A
Morassi et al. (46)	2020	Italy	CS	Appendix 3	16 Mar−5 April	Male (83.3); Female (16.7)	68.5 ± 10.6	6	N/A	N/A	5 (83.0)	N/A
Wang et al. (47)	2020	USA	CS	Appendix 3	N/A	Male (80); Female (20)	46 ± 10.2	5	N/A	N/A	3 (60.0)	N/A
Zayet et al. (48)	2020	France	CS	Appendix 3	25 Mar−3 April	Male (100)	79 ± 5	2	N/A	N/A	1 (50.0)	N/A
Fara et al. (49)	2020	USA	CS	Appendix 3	20 April	Male (20); Female (80)	55 ± 22	3	N/A	N/A	0 (0.0)	N/A
Valderrama et al. (21)	2020	USA	CR	Appendix 3	N/A	Male	52	1	N/A	N/A	Discharged	N/A
Viguier et al. (50)	2020	France	CR	Appendix 3	25 March	Male	73	1	N/A	N/A	Discharged	N/A
Sharafi Razavi et al. (51)	2020	Iran	CR	Appendix 3	N/A	Male	79	1	N/A	N/A	N/A	N/A
Christian Oliver et al. (52)	2020	Philippines	CR	Appendix 3	N/A	Female	62	1	N/A	N/A	N/A	N/A
Gonzalez-Pinto et al. (53)	2020	Spain	CR	Appendix 3	N/A	Female	36	1	N/A	N/A	Died	N/A
Moshayedi et al. (54)	2020	USA	CR	Appendix 3	N/A	Male	80	1	N/A	N/A	Died	N/A
Hughes et al. (55)	2020	UK	CR	Appendix 3	N/A	Male	59	1	N/A	N/A	Discharged	N/A
Gunasekaran et al. (56)	2020	USA	CR	Appendix 3	N/A	Female	40	1	N/A	N/A	Died	N/A
Goldberg et al. (57)	2020	USA	CR	Appendix 3	N/A	Male	64	1	N/A	N/A	N/A	N/A

*RC, Retrospective cohort; PC, Prospective cohort; CS, Case series; CR, Case report; N/A, Not applicable*.

* *Denotes study with characteristics all COVID-19 patients and not specific to stroke patients*.

#### Frequency of Stroke Among COVID-19 Patients

**Figure 2:**
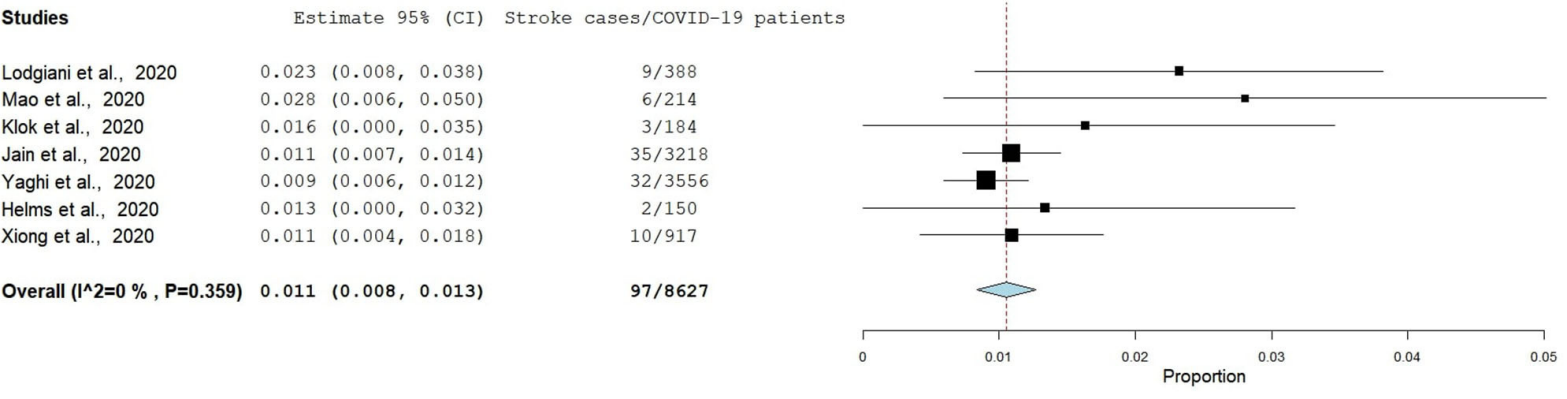
The forest plot of the pooled frequency of stroke among COVID-19 patients.

Eight studies had reported data eligible for the estimation of the pooled frequency of stroke among patients with COVID-19, and therefore the pooled frequency using the fixed-effect model is presented below in Figure 2. However, we decided to exclude the article by Benussi et al. in the final analysis due to its high heterogeneity. The pooled frequency of stroke among patients with COVID-19 as derived from the final seven studies was 1.1% (95% CI: 0.8, 1.3) and had a low degree of heterogeneity (*I*^2^ = 0.0%, *p* = 0.359) if the article by Benussi et al. (33) was excluded from the meta-analysis. The pooled frequency increased to 2.7% and heterogeneity was also extremely high (*I*^2^ = 96.3, *p* < 0.001) if the article by Benussi et al. (33) was included in the meta-analysis. Egger's test and Begg's test (*p* < 0.05) suggested that there was publication bias; sensitivity analysis also identified all seven studies in the meta-analysis had substantial influences on the pooled frequency of stroke among COVID-19 patients, which cause variation in a pooled frequency ranging from 1.0 to 1.2.

#### Data on Stroke Patients and Classification of Severity

The data on stroke patients in different groups of severe vs. non-severe COVID-19 infection and stroke patients with and without COVID-19 infection is shown in Table 2. Among the seven retrospective studies, two studies provided the number of patients having a stroke in the different groups of severe and non-severe COVID-19 infection (13, 32). One study reported the number of patients having a stroke in the different groups of with or without COVID-19 infection (33). Among patients who suffered from a stroke and classified according to the severity of the infection, the majority were placed in the severe COVID-19 infection group, whereby 60 patients were classified as severe compared to 29 in the non-severe group. Among patients who suffered from a stroke, 150 patients had COVID-19 infection, whereas 141 patients had no COVID-19 infection. The average days to develop stroke among patients after the onset of COVID-19 infection was 6.9 ± 4.5 days.

**Table 2:** Information on the number of stroke patients in groups with and without severe COVID-19 infection as well as with and without COVID-19 infection.

**References**	**No. of patients with stroke in groups with and without severe COVID-19 infection**				**Severity Stratification**	**When developed stroke, day in mean (SD, range or IQR)**	**No. of patients with stroke in groups of with or without COVID-19 infection**			
	**No. of stroke among patient with severe symptoms re**	**Total no. of patients with severe symptoms**	**No. of stroke among patients without severe symptoms**	**Total no. of patients in non-severe symptoms**			**No. of stroke among patients with COVID-19**	**Total no. of patients in COVID-19**	**No. of stroke among patients without COVID-19**	**Total no. of patients in non- COVID-19**
Lodgiani et al. (32)	3	61	6	327	ICU vs. General ward	N/A	9	388	N/A	N/A
Benussi et al. (33)	N/A	N/A	N/A	N/A	N/A	N/A	43	56	68	117
Klok et al. (34)	N/A	N/A	N/A	N/A	N/A	N/A	3	184	N/A	N/A
Jain et al. (35)	N/A	N/A	N/A	N/A	N/A	N/A	35	3218	N/A	N/A
Yaghi et al. (36)	26	N/A	6	N/A	American Thoracic Society for Community-Acquired Pneumonia	10 (range: 5–16.5)	32	3556	46	N/A
Escalard et al. (37)	N/A	N/A	N/A	N/A	N/A	6 (IQR 2–18)	10	N/A	27	N/A
Helms et al. (39)	N/A	N/A	N/A	N/A	ARDS	NA	2	150	N/A	N/A
Xiong et al. (40)	N/A	319	N/A	598	Republic of China Diagnosis and Treatment Protocol (Trial 6)	Late course (not specified)	10	917	N/A	N/A
Mao et al. (13)	5	88	1	126	American Thoracic Society for Community Acquired Pneumonia	9 (range: 1–18)	6	214	N/A	N/A
Beyrouti et al. (41)	5	Not applicable	1	Not applicable	Respiratory failure requiring ventilation	11.7 ± 7.9	N/A	N/A	N/A	N/A
Avula et al. (42)	3	Not applicable	1	Not applicable	Respiratory failure requiring ventilation	On admission D1	N/A	N/A	N/A	N/A
Zhang et al. (43)	3	Not applicable	0	Not applicable	Respiratory failure requiring ventilation	20.3 ± 11.7	N/A	N/A	N/A	N/A
Barios Lopez et al. (44)	2	Not applicable	2	Not applicable	ICU admission	7.25 ± 9.9	N/A	N/A	N/A	N/A
Oxley et al. (45)	N/A	N/A	N/A	N/A	N/A	N/A	N/A	N/A	N/A	N/A
Tunc et al. (Tunç et al., 2020)	0	Not applicable	4	Not applicable	Not applicable (all non-severe)	2 ± 1.2	N/A	N/A	N/A	N/A
Morassi et al. (46)	5	Not applicable	1	Not applicable	ARDS Severity	11.5 ± 0.7	N/A	N/A	N/A	N/A
Wang et al. (47)	3	Not applicable	2	Not applicable	NA	N/A	N/A	N/A	N/A	N/A
Zayet et al. (48)	1	Not applicable	1	Not applicable	Respiratory failure requiring ventilation	N/A	N/A	N/A	N/A	N/A
Fara et al. (49)	0	Not applicable	3	Not applicable	Not applicable (all non-severe)	On admission D1	N/A	N/A	N/A	N/A
Valderrama et al. (21)	Not applicable	Not applicable	1	N/A	Non-severe patient	7	N/A	N/A	N/A	N/A
Viguier et al. (50)	Not applicable	Not applicable	1	N/A	Non severe patient	7	N/A	N/A	N/A	N/A
Sharafi Razavi et al. (51)	N/A	N/A	N/A	N/A	N/A	3	N/A	N/A	N/A	N/A
Christian Oliver et al. (52)	Not applicable	Not applicable	1	Not applicable	Non-severe patient	14	N/A	N/A	N/A	N/A
Gonzalez-Pinto et al. (53)	1	Not applicable	Not applicable	Not applicable	Respiratory failure requiring ventilation	2	N/A	N/A	N/A	N/A
Moshayedi et al. (54)	1	Not applicable	Not applicable	Not applicable	Respiratory failure requiring ventilation	5	N/A	N/A	N/A	N/A
Hughes et al. (55)	Not applicable	Not applicable	1	Not applicable	Non-severe patient	4	N/A	N/A	N/A	N/A
Gunasekaran et al. (56)	1	Not applicable	Not applicable	Not applicable	Respiratory failure requiring ventilation	7	N/A	N/A	N/A	N/A
Goldberg et al. (57)	1	Not applicable	Not applicable	Not applicable	Respiratory failure requiring ventilation	16	N/A	N/A	N/A	N/A

*N/A, Not available*.

Regarding the severity stratification for COVID-19 infection, we observed that multiple stratification approaches were used across studies such as severe and non-severe infections that were based on admission to intensive care unit vs. general ward, the presentation of respiratory failure warranting intubation and ventilation, ARDS criteria, and according to guidelines from American Thoracic Society for community-acquired pneumonia as per Table 2.

#### Classification of Stroke Based on Imaging Findings in COVID-19 Patients

The imaging findings in COVID-19 patients are summarized in Table 3. Majority of strokes seen among COVID-19 patients were arterial stroke (98.5%) while venous stroke was seen only in three patients (1.5%). Ischemic stroke was the predominant stroke, and it was observed in 90.3% of stroke cases as compared to 9.7% patients presenting with hemorrhagic stroke. More than half of stroke happened in anterior circulation (60.0%), followed by the multiple territories (28.0%) and posterior circulation (12.0%). Among the 29 cases of stroke involving the anterior circulation, 28 cases occurred in middle cerebral artery (MCA) region, and only two cases involved the anterior cerebral artery (ACA) region.

**Table 3:** Classification of stroke based on imaging findings in COVID-19 patients.

**References**	**Arterial vs. Venous**, ***n***		**Ischemic vs. Hemorrhagic**, ***n***			**Location of stroke**, ***n***			
	**Arterial**	**Venous**	**TIA**	**Ischemic**	**Hemorrhagic**	**Anterior Circulation**		**Posterior circulation**	**Multiple territories**
						**MCA**	**ACA**		
Lodgiani et al. (32)	9	0	0	9	0	N/A	N/A	N/A	N/A
Benussi et al. (33)	43	0	5	35	3	N/A	N/A	N/A	N/A
Klok et al. (34)	3	0	0	3	0	N/A	N/A	N/A	N/A
Jain et al. (35)	35	0	0	26	9	N/A	N/A	N/A	N/A
Yaghi et al. (36)	32	0	0	32	0	N/A	N/A	N/A	N/A
Escalard et al. (37)	10	0	0	10	0	N/A	N/A	N/A	N/A
Helms et al. (39)	2	0	0	2	1	N/A	N/A	N/A	N/A
Xiong et al. (40)	10	0	0	10	3 (complication)	2	0	0	2 (N/A in 6 patients)
Mao et al. (13)	6	0	0	5	1	N/A	N/A	N/A	N/A
Beyrouti et al. (41)	6	2	0	6	0	2	0	1	3
Avula et al. (42)	4	0	0	4	0	4	0	0	0
Zhang et al. (43)	3	0	0	3	0	0	0	0	3
Barios Lopez et al. (44)	4	0	0	4	0	2	1	1	0
Oxley et al. (45)	5	0	0	5	0	4	0	1	0
Tunc et al. (58)	4	0	0	4	0	3	0	1	0
Morassi et al. (46)	6	0	0	5	1	0	1	1	2
Wang et al. (47)	5	0	0	5	0	4	0	1	0
Zayet et al. (48)	2	0	0	2	0	0	0	0	2
Fara et al. (49)	3	0	0	3	0	3	0	0	0
Valderrama et al. (21)	Yes	No	No	Yes	No	Yes	No	No	No
Viguier et al. (50)	Yes	No	No	Yes	No	Yes	No	No	No
Sharafi Razavi et al. (51)	Yes	No	No	No	Yes ^(LobarandSAH)^	Not applicable	Not applicable	Not applicable	Not applicable
Christian Oliver et al. (52)	Yes	No	No	Yes	No	Yes	No	No	No
Gonzalez-Pinto et al. (53)	Yes	No	No	Yes	No	Yes	No	No	No
Moshayedi et al. (54)	Yes	No	No	Yes	Yes ^(transformation)^	No	No	No	Yes (Anterior + posterior)
Hughes et al. (55)	No	Yes	No	Yes	No	N/A	N/A	N/A	N/A
Gunasekaran et al. (56)	Yes	No	No	Yes	No	Yes	No	No	No
Goldberg et al. (57)	Yes	No	No	Yes	No	No	No	No	Yes (MCA + bilateral ACA)

*TIA, Transient ischemic attack; MCA, Middle cerebral artery; ACA, Anterior cerebral artery; N/A, Not available; Pertaining to studies with only one patient with stroke, YesYes or No was used instead of n value*.

#### Classification of Stroke Based on Vessels Occlusion and TOAST Criteria in COVID-19 Patients

Table 4 summarized the stroke classification based on large vessels occlusion (LVO) and the TOAST (Trial of ORG 10172 in acute stroke treatment) classification (59) in patients with COVID-19. The numbers of stroke were almost equal for LVO (47 stroke cases in 10 studies) and non-LVO (42 cases in 10 studies). Location of LVOs involved were M1 vessels (21, 37, 41, 42, 44, 45, 47, 53, 54), M2 vessel (41, 42, 44, 45, 47, 53), internal carotid (21, 37, 42, 45, 47, 53), multiteritorial (37, 47), posterior cerebral (41, 45), basilar (37), ACA (21, 53), and the vertebral artery (41).

**Table 4:** Classification of stroke based on vessels occlusion and TOAST in COVID-19 patients.

**References**	**Large vessels occlusion (LVO) vs non-LVO**		**Location of large vessels, *n***	**Classification of stroke based on TOAST**, ***n***				
	**LVO**	**Non-LVO**		**Large vessels**	**Small vessel**	**Cardioembolic**	**Cryptogenic**	**Others**
Lodgiani et al. (32)	N/A	N/A	N/A	N/A	N/A	N/A	N/A	N/A
Benussi et al. (33)	Not applicable	Not applicable	Not applicable	Not applicable	Not applicable	Not applicable	Not applicable	Not applicable
Klok et al. (34)	N/A	N/A	N/A	N/A	N/A	N/A	N/A	N/A
Jain et al. (35)	N/A	N/A	N/A	17	9	0	0	0
Yaghi et al. (36)	45.5%	55.50%	N/A	2	0	7	21	2
Escalard et al. (37)	100% (they include only LVO)	0	Carotid terminus (30%); M1 (60%), M2 (0%), Basillar (10%), Multiteritorial (50%)	N/A	N/A	N/A	N/A	N/A
Helms et al. (39)	N/A	N/A	NA	N/A	NA	N/A	N/A	N/A
Xiong et al. (40)	N/A	N/A	N/A	N/A	N/A	N/A	N/A	N/A
Mao et al. (13)	N/A	N/A	N/A	N/A	N/A	N/A	N/A	N/A
Beyrouti et al. (41)	6	0	M1 (1), M2 (1), Posterior cerebral (2), Vertebral (1), Unknown (1)	NA	NA	NA	NA	NA
Avula et al. (42)	2	2	Internal carotid (1), MCA 1 (not specified M1 or M2)	NA	NA	NA	NA	NA
Zhang et al. (43)	0	3	Not applicable - Non LVO	0	0	0	0	0
Barios Lopez et al. (44)	1	2 (1 not known)	MCA 1 (not specified, M1 or M2)	0	0	2	0	0
Oxley et al. (45)	5	0	Internal Carotid (1), MCA 3 (not specified M1 or M2), Posterior cerebral (1)	NA	NA	NA	NA	NA
Tunc et al. (Tunç et al., 2020)	0	4	Not applicable—Non LVO	2	2	0	0	2
Morassi et al. (46)	0	6	Not applicable—Non LVO	N/A	N/A	N/A	N/A	N/A
Wang et al. (47)	5	0	Internal carotid (2), M1 (1), Tandem carotid+M2 (1), Multiteritorial (1)	NA	NA	NA	NA	NA
Zayet et al. (48)	0	2	Not applicable—Non-LVO	N/A	N/A	N/A	N/A	N/A
Fara et al. (49)	0	3	Not applicable—Non LVO	3	0	0	0	3
Valderrama et al. (21)	Yes	No	Internal carotid + MCA (proximal M1) and ACA	Yes	No	No	No	No
Viguier et al. (50)	No	Yes	Not applicable (non-occlusive ICA thrombus)	Yes (ICA)	No	No	No	No
Sharafi Razavi et al. (51)	Not applicable	Not applicable	Not applicable (because bleeding)	No	No	No	No	No
Christian Oliver et al. (52)	No	Yes	Not applicable (Non-LVO)	Yes (M1)	No	No	No	No
Gonzalez-Pinto et al. (53)	Yes	No	Internal carotid + MCA and ACA	Yes	No	No	No	No
Moshayedi et al. (54)	Yes	No	Proximal M1	No	No	Yes	No	No
Hughes et al. (55)	Not applicable	Not applicable	Not applicable (because cerebral venous thrombosis -sigmoid and transverse sinus	N/A	N/A	N/A	N/A	N/A
Gunasekaran et al. (56)	N/A	N/A	NA	N/A	N/A	N/A	N/A	N/A
Goldberg et al. (57)	No	Yes	Not applicable (Non-LVO)	Yes	No	No	No	No

*LVO, Large vessels occlusion; MCA, Middle cerebral artery; ACA, Anterior cerebral artery; N/A, Not available; For LVO—cohort % but case series number; Pertaining to studies with only one patient with stroke, Yes or No was used instead of n value*.

According to the classification of stroke based on the TOAST criteria, we found that large vessels and cryptogenic were the most common type of stroke (28.9%), followed by cardioembolic (15.7%), small vessels (14.0%), and others (12.4%). A majority of the studies did not classify their stroke type with the TOAST classification.

#### Comorbidities Among Patients in the Study

Table 5 shows the data on comorbidities among participants in the included studies. Hypertension (50.9%) was found to be the highest in percentage among the comorbidities, followed by diabetes (40.0%), atrial fibrillation (23.9%), hyperlipidaemia (17.0%), history of ischemic heart disease (14.8%), smoking (10.5%), previous stroke (6.7%), malignancy (4.5%), chronic kidney disease or end-stage renal disease (2.9%), and finally heart failure (0.4%).

**Table 5:** Information on comorbidities and smoking habit among patients with COVID-19 infection in the study.

**References**	**Comorbidities, %**									
	**Diabetes**	**Hyperlipidaemia**	**Hypertension**	**Ischemic heart disease**	**Heart Failure**	**Previous stroke**	**Chronic kidney disease/ end-stage renal disease**	**Malignancy**	**Atrial Fibrillation**	**Smoking**
Lodgiani et al.^*^ (32)	32.7	19.6	47.2	13.9	N/A	5.2	15.7	6.4	Unclear	11.6
Benussi et al. (33)	23.3	23.3	60.5	18.6	N/A	N/A	4.7	11.6	N/A	2.3
Klok et al.^*^ (34)	N/A	N/A	N/A	N/A	N/A	N/A	13	2.7	N/A	N/A
Jain et al.^*^ (35)	N/A	N/A	37.9	N/A	N/A	N/A	N/A	N/A	N/A	N/A
Yaghi et al. (36)	34.4	56.3	56.6	15.6	6.3	3.1	N/A	N/A	18.8	0
Escalard et al. (37)	40	30	50	N/A	N/A	N/A	N/A	N/A	10	10
Helms et al.^*^ (39)	20	N/A	N/A	48	N/A	4.7	4	6	20	N/A
Xiong et al. (40)	N/A	N/A	N/A	N/A	N/A	N/A	N/A	N/A	N/A	N/A
Mao et al.^*^ (13)	14	N/A	23.8	7	N/A	N/A	2.8	6.1	N/A	N/A
Beyrouti et al. (41)	33.3	0	66.7	33.3	0	16.7	0	33.3	33.3	16.7
Avula et al. (42)	25	75	100	0	0	0	25	0	0	Not mentioned
Zhang et al. (43)	75	0	100	33.3	0	66.7	0	33.3	0	Not mentioned
Barios Lopez et al. (44)	75	0	50	50	0	0	0	0	25	25
Oxley et al. (45)	20	20	20	0	0	20	0	0	0	0
Tunc et al. (58)	25	0	75	0	0	0	0	0	0	0
Morassi et al. (46)	50	0	66.7	16.7	N/A	16.7	0	0	0	16.7
Wang et al. (47)	20	0	20	40	0	0	0	0	0	0
Zayet et al. (48)	100	0	50	50	0	0	0	0	100	0
Fara et al. (49)	33.3	33.3	33.3	0	0	0	0	0	0	Not mentioned
Valderrama et al. (21)	No	No	Yes	No	No	No	No	No	No	No
Viguier et al. (50)	No	No	No	No	No	No	No	No	No	No
Sharafi Razavi et al. (51)	N/A	N/A	N/A	NA	NA	NA	NA	NA	N/A	N/A
Christian Oliver et al. (52)	Yes	Yes	Yes	No	No	No	No	No	No	No
Gonzalez-Pinto et al. (53)	No	No	No	No	No	No	No	No	No	No
Moshayedi et al. (54)	N/A	N/A	N/A	N/A	N/A	N/A	N/A	N/A	N/A	N/A
Hughes et al. (55)	Yes	No	Yes	No	No	No	No	No	No	No
Gunasekaran et al. (56)	Yes	No	No	No	No	No	No	No	No	No
Goldberg et al. (57)	No	No	Yes	No	No	No	No	No	No	No

*N/A, Not available*.

* *Denotes study with characteristics all COVID-19 patients and not specific to stroke patients; Pertaining to studies with only one patient with stroke, Yes or No was used instead of n value*.

#### Blood Parameters Among COVID-19 Patients Included in Study

Daa on the blood parameters are shown in Table 6. Functions of each of the blood tests and its normal range are summarized in Appendix 4. The mean for erythrocyte sedimentation rate (ESR) was in a range of 31–86 mm/1 h. For C-reactive protein (CRP), the mean ranged from 0.101 to 1,920 mg/L, in which majority of studies had a CRP results exceeding the normal range except for the study by Yaghi et al. (36), in which the CRP reading was 0.101 mg/L. We observed that almost all studies had elevated ferritin readings that ranged from 392 to 4609.33 mg/L, except for a study done by Tunc et al. (58), in which the ferritin level was 150.5 mg/L. For lactic acid dehydrogenase (LDH) test, elevated LDH readings across the studies were observed that ranged from 406 to 860.4 IU/L, except for normal levels seen in studies done by Mao et al. (241.5 IU/L) (13) and Benussi et al. (275.7 IU/L) (33).

**Table 6:** Blood parameters as inflammation markers among COVID-19 patients with stroke.

**References**	**Blood parameters as inflammation markers**										**Platlet (x10^**9**^)**	**PT (secs)**
	**ESR (mm/ 1hr)**	**CRP (mg/L)**	**Ferritin (mg/L)**	**D-dimer (mg/L)**	**LDH (IU/L)**	**Fibrinogen (mg/dL)**	**Antiphospholipids**	**Procalcitonin, ng/mL**	**IL-6 (pg/mL)**	**Troponin (pg/mL)**		
Lodgiani et al.^*^ (32)	N/A	N/A	N/A	Day 4–6: Total survivor 0.389 (0.246–0.685)Day 4–6: Total non-survivor 0.943 (0.611–2.618)	N/A	N/A	N/A	N/A	N/A	N/A	N/A	N/A
Benussi et al. (33)	49 (range 29.3-85)	28 (range 44-71.1)	452.5 (range 268.8-871)	735.5 (range 364.3–2910.8)	277.7 (range 239 - 365)	471 (range 368-545)	N/A	N/A	N/A	27.0 (range 10.0–41.0)	276 (range 197–315) 264.3)	12.8 (range 12.2– 15.6)
Klok et al. (34)	N/A	N/A	N/A	N/A	N/A	N/A	N/A	N/A	N/A	N/A	N/A	N/A
Jain et al. (35)	N/A	N/A	N/A	N/A	N/A	N/A	N/A	N/A	N/A	N/A	N/A	N/A
Yaghi et al. (36)	79 (IQR 53)vs 40 (IQR86)^*^	0.101 (IQR 0.176)	N/A	3.9 (IQR 7.4)	N/A	N/A	N/A	N/A	Only in 8 patients 10.5 (IQR 96.25) in covid positive	N/A	N/A	N/A
Escalard et al. (37)	N/A	N/A	N/A	N/A	N/A	N/A	N/A	N/A	N/A	N/A	N/A	N/A
Helms et al.^*^ (39)	N/A	N/A	N/A	2.27 (IQR 1.16 - 20)	N/A	699 (IQR 608 - 773)	Positive Lupus anticoagulants in 50 patients	N/A	N/A	N/A	200 (IQR 152–267)	84 (73–91)
Xiong et al. (40)	N/A	N/A	N/A	N/A	N/A	N/A	N/A	N/A	N/A	N/A	N/A	N/A
Mao et al.^*^ (13)	N/A	12.2 (range 0.1 - 212)	N/A	0.5 (range 0.1 - 20)	241.5 (range 2.2 - 908)	N/A	N/A	N/A	N/A	N/A	209 (range 18–583)	N/A
Beyrouti et al. (41)	N/A	139.5 ± 108.0	1925.8 ± 1732.9	25.3 ± 28.3	502.5 ± 127.0	628 ± 202	5 patients: Positive lupus anticoagulant and 1 patient: Positive medium titer IgM anti-cardiolipin + low-titer IgG and IgM anti–β2-glycoprotein-1	NA	NA	31.1 ± 21.7 High sensitive pg/mL	290 ± 100.6	15.5 ± 9.3
Avula et al. (42)	N/A	184 ± 57 (in 3 patients)	513.5 (135.9 - 891) ng/L	8.704 ± 5.62 (in 2 patients)	456 ± 256	N/A	N/A	4.9 ± 6.5 (in 2 patients)	8.5 pg/mL (1 patient)	0.065 (<0.01–0.14) pg/mL	219 ± 94.5	15.5 ± 2.5
Zhang et al. (43)	N/A	97.8 ± 36.8	2207.8 (only in 1 patient)	9.0 ± 10.4	427 ± 199.7	500 ± 120	3 patients: positive for IgA anti-cardiolipin and IgA and IgM anti–β2-glycoprotein-1	0.23 ± 1.6	NA	1338.9 ± 2198.7 pg/mL (troponin I)	112 ± 58.6	16.4 ± 1.2
Barios Lopez et al. (44)	N/A	82.6 ± 115.4	722.6 ± 590.5	28.5 ± 44.3	508.8 ± 283	462.8 ± 113.2	1 patient: positive IgG anti–β2-glycoprotein-1	0.28 ± 0.36	3 pg/mL	17.9 ± 11.8 pg/mL	293 ± 6.9	12.2 ± 0.8
Oxley et al. (45)	N/A	N/A	659 ± 638.2	3.66 ± 5.8	N/A	516.8 ± 138.6	N/A	N/A	N/A	N/A	297 ±112.4	13.8 ± 1.0
Tunc et al. (Tunç et al., 2020)	N/A	N/A	150.5 ± 68.7	0.713 ± 0.24	N/A	N/A	N/A	N/A	N/A	N/A	N/A	N/A
Morassi et al. (46)	N/A	93.6 ± 94	N/A	3.9 ± 3.3	860.4 ± 223.4	N/A	N/A	N/A	N/A	N/A	N/A	N/A
Wang et al. (47)	Elevated	Elevated	N/A	Elevated	N/A	N/A	N/A	N/A	Elevated	N/A	N/A	Elevated
Zayet et al. (48)	N/A	69.5 ± 39.5	N/A	10278.5 ± 8092.5 (out of range despite same unit)	505.5 ± 179.5	6,050 ± 550	1 patient: positive IgM anti-cardiolipin	N/A	NA	791.3 ± 744.7	171.5 ± 98.5	12.05 ± 0.25
Fara et al. (49)	N/A	Elevated in all patients	N/A	Elevated in 1 patient	N/A	NA	1 patient: positive low titer IgM anti-cardiolipin	N/A	N/A	N/A	N/A	N/A
Valderrama et al. (21)	37	11	588	>10	N/A	235	NA	N/A	NA	N/A	N/A	N/A
Viguier et al. (50)	N/A	219	1096	2.2	N/A	820	Negative	N/A	N/A	N/A	N/A	N/A
Sharafi Razavi et al. (51)	85	10	NA	NA	N/A	NA	NA	N/A	N/A	N/A	210	12
Christian Oliver et al. (52)	86	1920	4609.33	1.16	406	NA	NA	0.8 ng/mL	NA	145 pg/mL	409	12.9
Gonzalez-Pinto et al. (53)	N/A	N/A	N/A	7.54	N/A	N/A	N/A	N/A	N/A	N/A	NA	N/A
Moshayedi et al. (54)	N/A	N/A	N/A	NA	N/A	N/A	N/A	N/A	N/A	N/A	NA	N/A
Hughes et al. (55)	31	15	N/A	NA	N/A	Unclear	N/A	N/A	N/A	N/A	202	11.1
Gunasekaran et al. (56)	N/A	N/A	3079	28.07 ng/ml	N/A	860	Negative	N/A	N/A	N/A	303	N/A
Goldberg et al. (57)	N/A	N/A	N/A	N/A	N/A	N/A	Positive IgM anti-cardiolipin	N/A	N/A	N/A	N/A	N/A

*ESR, erythrocyte sedimentation rate; CRP, C-Reactive Protein; LDH, Lactic Acid Dehydrogenase; IL-6, Interleukin 6; PT, prothrombin time; N/A, Not available*.

* *Denotes study with characteristics all COVID-19 patients and not specific to stroke patients*.

A majority of the studies included had an elevated mean for the D-dimer test, which ranged from 0.71 to 28.5 mg/L except for the study done by Lodigiani et al. (32), in which the mean of D-dimer was 0.389 on day 4–6 among the survivors, and an elevated D-dimer of 0.943 was reported among the non-survivors.

For the fibrinogen test, a majority of studies reported that the mean for fibrinogen was out of the normal range (200–400 mg/dL), in which they ranged from 462.8 to 6,050 mg/dL, except for the study done by Valderrama et al., which had a normal level (235 mg/dL) (21).

Similarly, a majority of the studies did not capture information on the presence of antiphospholipid, except the studies by Helms et al. (39), Beyrouti et al. (41), Zhang et al. (43), Barios Lopez et al. (44), Zayet et al. (48), Fara et al. (49), and Goldberg et al. (57), in which these studies reported positive findings for the presence of antiphospholipid antibodies. On the other hand, studies by Viguier et al. (50) and Gunasekaran et al. (56) reported the absence of antiphospholipid antibodies. For the procalcitonin titres, three studies had a blood test result of below 1.0 mg/mL, which ranged from 0.23 to 0.8 ng/mL (43, 44, 52), with the highest mean for procalcitonin concentration reported in the study by Avula et al. (4.9 ng/mL) (42). We observed that only three studies captured information on interleukin-6 (IL-6) levels among patients with COVID-19 and stroke, which ranged from 3 to 10.5 pg/mL, which are the studies by Avula et al. (42), Barios Lopez et al. (44), and Yaghi et al. (36). Data of all these studies reported a normal reading for IL-6 levels. For the troponin test, seven studies reported data on the troponin concentration (33, 41–44, 48, 52). Three out of the seven studies reported an abnormally elevated troponin concentration, which were 1338.9 pg/mL (43), 791.3 pg/mL (48), and 145 pg/mL, respectively (52). For the prothrombin time, a majority of studies reported levels that fell in the normal range (11–13.5 s) except for the studies by Helmes et al. (39), Zhang et al. (43), Avula et al. (42), and Oxley et al. (45).

For the platelet level, the mean ranged from 112 to 303 × 10^9^, and the levels were all within the normal range in the included studies, except for the study done by Christian Oliver et al. (52), which had a slightly elevated level (409 × 10^9^). The normal range for clotting time (prothrombin test) is 11–13.5 s (60). Among the included studies, six studies reported a normal mean for the prothrombin time, and these studies included the studies by Benussi et al. (33), Barios Lopez et al. (44), Zayet et al. (48), Sharafi-Razavi et al. (51), Christian Oliver et al. (52), and Hughes et al. (2020). Five studies reported an abnormal mean for the prothrombin time, with a prothrombin time of 13.8 s for the study by Oxley et al. (45), 15.5 s for Beyrouti et al. (41) and Avula et al. (42), 16.4 seconds for Zhang et al. (43), and up to 84 s for the study by Helms et al. (39).

## Discussion

The aim of this current study is to perform a systematic review and meta-analysis concerning the epidemiological, clinical presentation, imaging characteristics, and laboratory findings related to both stroke and COVID-19 infection.

### SARS-CoV-2 Features, Epidemiological Findings, and Its Comorbidities in Stroke Patients With COVID-19

Coronaviruses are divided into four genera, in which the new coronavirus (SARS-CoV-2) is classified into the beta genus, which includes viruses causing SARS and MERS (Middle East Respiratory Syndrome) as well (61). There are now at least seven human coronaviruses, including SARS-CoV-1, SARS-CoV-2, MERS-CoV, HCoV-OC43, HCoV-229E, HCoV-NL-63, and HCoV-HKU1 (38). Studies on previous human coronaviruses infections indicated that the virus does not remain confined to the respiratory system and may also disseminate to other organs, including the central nervous system via the angiotensin-converting enzyme type 2 receptor (ACE-2) (62, 63). The possibility of neurological complications may stem from the neurotropic and neurovirulent property of SARS-CoV-2, which are also seen in other human coronaviruses (64).

The association of stroke with viral infection is well-established, albeit uncommon. In general, viral infection, particularly those in the early convalescence phase, increases the odds of stroke by 1.4-folds (17). A previous study amongst SARS-COV-1 patients showed that LVO occurred in a small percentage of patients (2.4%) that were infected in which the two patients had cardiac dysfunction, disseminated intravascular coagulation, and significant hypotension before the onset of stroke (65). A similar trend among MERS patients also showed that only a small number of patients developed stroke associated with preceding disseminated intravascular coagulation in one of the patients (66).

In this current review, the pooled frequency of stroke was 1.1%. We decided to remove Benussi et al. (33) in the final result as the study was conducted in a stroke hub for COVID-19 in Italy, which explained the high frequency of stroke (76.8%) among patients with COVID-19. We found that overall, patients with COVID-19 exhibited a lower percentage of stroke, which was 1.1% of patients with COVID-19. This is similar to the worldwide prevalence of stroke (1.12%) (67) but much lower as compared to the prevalence of stroke in the United States (2.5%) and in China (3.1%) (68, 69). The association of stroke seen in patients with COVID-19 may be attributed to the shared traditional risk factors for stroke also seen in COVID-19 patients. Literature reported that the traditional risk factors for stroke are diabetes, hypertension, hyperlipidemia, smoking, atrial fibrillation, previous stroke, ischemic heart disease, and family history of stroke, in which the estimated relative risk for total stroke associated with hypertension was 5.43 (70), 2.28 for diabetes (71), 1.64 for obesity (72), 1.46 for atrial fibrillation (73), and 1.10 for chronic kidney disease (74). Our finding is consistent with the literature that reported that more than half of COVID-19 patients with stroke had comorbidities of hypertension, followed by diabetes, atrial fibrillation, hyperlipidaemia, and/or history of ischemic heart disease.

### Imaging Characteristics of Stroke in COVID-19 Infection

Ischemic stroke is the most common type of stroke seen in this review as compared to less frequently occurring haemorrhagic and transient ischemic stroke. Hypertension, diabetes, and cardiovascular disease are known risk factors for ischemic stroke (75). In addition, the risk factors of hemorrhagic and ischemic strokes were also relatively similar (INTERSTROKE study). A recent review showed that all infections increase the risk of acute ischemic stroke, although its pathophysiology is not adequately explained (76).

Anterior circulation is the most common site for stroke, with more than half of the strokes occurring in the middle cerebral artery, followed by the multiple territories. Interestingly in our review, a quarter of the stroke was multi-territorial. This may be due to the propensity of systemic embolisation and microvascular thrombosis that typically occurs in COVID-19 infection due to the excessive production of prothrombotic factors and dysregulation of the anti-thrombotic properties (77), whereas strokes are less commonly seen in the posterior and anterior cerebral arteries (78). This observation is similar to the non-COVID-19 related stroke.

A recent report pointed out the propensity of LVO to occur in patients with COVID-19 and its tendency to occur in the younger age group (45). In our review of the currently available literature, half of the reported stroke cases were due to an LVO as compared to non-large vessel occlusion. This rate is much higher as compared to the general population where LVO usually occurs in around one-third of the patients (79). Furthermore, among studies that used the TOAST classification, one-third reported stroke types as cryptogenic and others that indicate that there are other underlying pathologies apart from the traditional risk factors that contribute to the occurrence of stroke in patients with COVID-19.

### Laboratory Finding and Its Association With the Pathophysiology of Stroke Patients With COVID-19

Apart from the possible neuropathic property of SARS-CoV-2 that causes direct endothelial injury via the ACE-type 2 receptor (80) and sharing of the common traditional risk factors for stroke, the pathophysiology of stroke in COVID-19 patients could also be attributed to the pro-inflammatory and hypercoagulable state predisposing to thrombosis. The thrombo-inflammatory nature of SARS-CoV-2 was noted as to be associated with elevated levels of D-dimer, fibrinogen, platelet, and IL-6 (77). Furthermore, the excessive systemic immune response that may be seen in this novel infection may be due to immunopathogenicity in which the over-stimulation of the immune system by this virus leads to attacks to one's own immune system (81). Cytokine storm may also occur as our immune system goes into an overdrive, leading to a massive influx of SARS-related inflammatory cytokine such as interleukin-1β, IL6, IL12, interferon-γ, inducible protein−10, and monocyte chemoattractant protein-1 (81, 82). These excessive inflammatory cascades may lead to two main sequelae [i.e., production of prothrombotic factors and endothelium damage due to dysregulation of anti-thrombotic properties, subsequently leading to microvascular thrombosis with potential for systemic embolization (77, 83)]. Moreover, inflammatory markers [e.g., C-reactive protein and fibrinogen, are independent risk factors for ischemic stroke and may also predispose to atherosclerosis and endothelial dysfunction that can be further exacerbated by infection (81, 84)].

Hypercoagulable state, on the other hand, as demonstrated by elevated D-dimer levels, abnormality in clotting variables, and hyperferritinemia, not only increases the risk of a thromboembolic event but is also an independent predictor for poor prognosis and mortality (4, 40). The role of other thrombotic markers such as the antiphospholipid antibodies, albeit their role in COVID-19, are also uncertain but may also contribute to the hypercoagulable state (43).

In our review, several markers are commonly used to identify the thrombo-inflammatory nature of COVID-19 (e.g., D-dimers, CRP, ferritin, fibrinogen, antiphospholipid antibodies, LDH, and troponin). Based on our observation, CRP was the most commonly used biomarker, followed by D-dimer, LDH, troponin, and antiphospholipid tests. In this review, stroke patients with COVID-19 consistently presented with an elevated level of D dimers, CRP, ferritin, LDH, troponin, ESR, fibrinogen, and with positive antiphospholipid antibodies reported in some studies. IL-6 and pro-calcitonin were only reported in a few studies and were not found to be elevated.

### Subgroup Analysis on Characteristics of Stroke Patients With and Without COVID-19 Infection

Although the mean age of patients with COVID-19 and stroke in our review was 62.9 years, many case series and case reports have shown that those in the younger age group or those with no comorbidities more commonly presented with stroke (42, 43, 49, 50, 53, 56, 58). Furthermore, stroke is shown to occur early in the illness with mean onset at 6.9 days, with reports even showing that patients may present with stroke and at the same time have asymptomatic COVID-19 infection (42, 49). Unfortunately, patients with COVID-19 and stroke had a more severe COVID-19 infection and a poorer prognosis with a higher mortality rate as shown by this current review. The mean mortality rate among stroke patients with COVID-19 infection was 46.7% compared to only 8.7% among those without COVID-19 infection, and this could be attributed to the severity of infections in patients concurrently having neurological complications (13, 53, 54, 56).

A subgroup analysis was done among the population cohorts of the Benussi et al., Yaghi et al., and Escalard et al. studies, which had data on both stroke patients with and without COVID-19 infection (Table 7). The cohorts in Yaghi et al. and Escalard et al. studies had more males and younger patients. In contrast, similar age and gender characteristics were seen in the study by Benussi et al. All three cohorts showed the presence of traditional cardio-cerebrovascular comorbidities in patients with COVID-19 infection, which may contribute to the pathophysiology of the stroke. Furthermore, more LVOs were seen in patients with COVID-19 in the study by Yaghi et al. (i.e., 45.5 vs. 27.9%), while the cohort in the study by Escalard et al. only included patients with LVO. In the Yaghi et al. cohort, more cryptogenic strokes were reported among the patients with COVID-19, which required further investigations on its unusual etiology. Interestingly, more hemorrhagic stroke was seen in non-COVID infected patients in the study by Benussi et al., which may suggest the possibility of a thrombotic phenomenon in large vessels that are more predominant in COVID-19 infections rather than the small vessel disease leading to the occurrence of hemorrhage. Mortality was also higher in all three cohorts among patients with COVID-19 infection [i.e., in the studies by Benussi et al. (34.9 vs. 5.9%), Yaghi et al. (63.6 vs. 9.3%), and Escalard et al. (60.0 vs. 11.0%), respectively]. Given the high mortality rates associated with stroke in patients with COVID-19 infection that may cause a more severe stroke with an LVO, future studies are required to investigate the stroke characteristics among patients with COVID-19.

**Table 7:** Subgroup analysis on characteristics of stroke patients with and without COVID-19 infection.

**References**	**Male,%**		**Female, %**		**Age was presented either in mean** **±** **standard deviation or median (Interquartile range), or mean (range)**		**Comorbidities and smoking habit**																			**Mortality rate, %**	
							**Diabetes, %**		**Hyperlipidemia, %**		**Hypertension, %**		**Ischemic heart disease, %**		**Heart failure, %**		**Previous stroke, %**		**Chronic kidney disease/ end-stage renal disease, %**		**Malignancy, %**		**Atrial fibrillation, %**		**Smoking, %**			
	**Stroke C+**	**Stroke C-**	**Stroke C+**	**Stroke C-**	**Stroke C+**	**Stroke C-**	**Stroke C+**	**Stroke C-**	**Stroke C+**	**Stroke C-**	**Stroke C+**	**Stroke C-**	**Stroke C+**	**Stroke C-**	**Stroke C+**	**Stroke C-**	**Stroke C+**	**Stroke C-**	**Stroke C+**	**Stroke C-**	**Stroke C+**	**Stroke C-**	**Stroke C+**	**Stroke C-**	**Stroke C+**	**Stroke C-**	**Stroke C+**	**Stroke C-**
Benussi et al. (33)	51.2	58.8	48.8	41.2	75.9 (range = 66.8–85.2)	74.8 (range = 58.3–80.4)	23.3	20.6	23.3	33.8	60.5	75	18.6	11.8	N/A	N/A	N/A	N/A	4.7	4.4	11.6	27.9	N/A	N/A	2.3	8.8	34.9	5.9
Yaghi et al. (36)	71.9	52.2	28.1	48.8	63 (IQR=17)	70 (IQR = 18)	34.4	28.3	56.3	50	56.6	76.1	15.6	26.1	6.3	10.9	3.1	13.0	N/A	N/A	N/A	N/A	18.8	21.7	0	4.3	63.6	9.3
Escalard et al. (37)	80	48	20	52	59.5 (range = 54)	72 (range = 60–81.5)	40	22	30	37	50	55	N/A	N/A	N/A	N/A	N/A	N/A	N/A	N/A	N/A	N/A	10	30	10	26	60	11

*Stroke C+: Stroke patients with COVID-19 infection; Stroke C-: Stroke patients without COVID-19 infection. N/A: Not available*.

### Clinical Implications

Although COVID-19 may predominantly present with respiratory symptoms, this review may create awareness among clinicians on potential presentation of stroke in those having this infection, especially for those with severe infection. As many of the patients share similar traditional risk factors for stroke, the presentation of a patient with stroke to the emergency department in this current pandemic must be reviewed cautiously and treated with high suspicion of the potential presence of SARS-CoV-2 infection in order to prevent further dissemination and deterioration. The role of specific blood tests as a potential thrombo-inflammatory marker can be a guide to predict the possible thromboembolic occurrence and disease severity, hence providing much-needed guidance for physicians in taking necessary preventative measures.

### Strength and Limitations

This is the first systematic review summarizing the findings in relation to both COVID-19 and stroke. We found a high incidence of stroke among patients with COVID-19. The majority are ischemic stroke, involve large vessels occlusion, and occurs predominantly in the middle cerebral artery. We also found hypertension as the most common comorbidity among this study participants. Most of the laboratory tests except for IL-6 and procalcitonin appeared to be useful for indicating the presence of inflammation and the prothrombotic state as a predictor for stroke, although results varied between the studies.

This review has several limitations. First, the majority of studies did not provide data based on the severity of the infection, and therefore meta-synthesis for severe cases of COVID-19 and the risk of stroke cannot be performed with the existing studies. Similarly, it is impossible to meta-synthesize the risk of stroke associated with COVID-19 infection for all studies due to the lack of data on stroke characteristics among non-COVID-19 patients. Second, due to the lack of data of comorbidities for participants in the control group, analysis of the associated factors for stroke cannot be performed for this review.

Third, we also found that many varied types of blood tests were used for identifying inflammation and hypercoagulable state; thus, the usefulness of laboratory tests results in identifying patients with high risk for stroke could not be determined with the existing literature. Future research with bigger sample size is needed to rectify these important issues.

## Conclusion

The occurrence of stroke in patients with COVID-19 infection is uncommon but poses as an important prognostic marker and severity indicator. This brief review suggests that ischemic stroke may occur early in the course of the illness, and may also affect patients in the younger age groups with no comorbidities, causing large vessel occlusion and exhibiting thrombo-inflammatory vascular picture. Given that many patients with COVID-19 share the common traditional risk factors for stroke, physicians must be vigilant in the future for an increase in the number of strokes in patients with COVID-19 as the pandemic continues and to take appropriate preventive measures.

## Author Contributions

AK, SC, WL, LM, WS, HB, and FH: conceptualization. AK, PC, WL, AR, JB, LM, ND, and DS: data curation. KL, AK, SC, PC, AR, and ND: formal analysis. AK, SC, PC, AR, JB, LM, HB, and FH: investigation. KL, SC, PC, WL, AR, and JB: methodology. AK and WS: project administration. KL: software. HB and FH: supervision. PC, JB, and DS: validation. DS: visualization. KL, AK, SC, WS, ND, and FH: writing—original draft. KL, AK, SC, PC, WL, AR, JB, LM, WS, ND, HB, and FH: writing—review and editing. All authors contributed to the article and approved the submitted version.

## Conflict of Interest

The authors declare that the research was conducted in the absence of any commercial or financial relationships that could be construed as a potential conflict of interest.

## Abbreviations

ACA: Anterior cerebral artery
ACE-2: Angiotensin-converting enzyme type 2 receptor
ARDS: Acute respiratory distress syndrome
CI: Confidence interval
CR: Case report
CRP: C-reactive protein
CS: Case series
ESR: Erythrocyte sedimentation rate
IL-6: Interleukin 6
LDH: Lactic acid dehydrogenase
LVO: Large vessels occlusion
MCA: Middle cerebral artery
PRISMA: Preferred Reporting Items for Systematic Reviews and Meta-Analyses
PT: Prothrombin time
RC: Retrospective cohort
TIA: Transient ischemic attack
TOAST: Trial of ORG 10172 in acute stroke treatment
WHO: World Health Organization.
