# Supplementary material for: Stroke and Novel Coronavirus Infection in Humans: A Systematic Review and Meta-Analysis
Source: Front Neurol. 2020 Oct 6;11:579070. doi: 10.3389/fneur.2020.579070 (PMC7573135; doi:10.3389/fneur.2020.579070)
Supplement: Supplementary file 1 [file Data_Sheet_1.docx]

| Appendix 1: PRISMA checklist for cohort studies | | | |
| --- | --- | --- | --- |
| **Section/topic** | **#** | **Checklist item** | **Reported on page #** |
| **TITLE** | | |  |
| Title | 1 | Identify the report as a systematic review, meta-analysis, or both. | 1 |
| **ABSTRACT** | | |  |
| Structured summary | 2 | Provide a structured summary including, as applicable: background; objectives; data sources; study eligibility criteria, participants, and interventions; study appraisal and synthesis methods; results; limitations; conclusions and implications of key findings; systematic review registration number. | 3 |
| **INTRODUCTION** | | |  |
| Rationale | 3 | Describe the rationale for the review in the context of what is already known. | 4 |
| Objectives | 4 | Provide an explicit statement of questions being addressed with reference to participants, interventions, comparisons, outcomes, and study design (PICOS). | 4 |
| **METHODS** | | |  |
| Protocol and registration | 5 | Indicate if a review protocol exists, if and where it can be accessed (e.g., Web address), and, if available, provide registration information including registration number. | 5 |
| Eligibility criteria | 6 | Specify study characteristics (e.g., PICOS, length of follow-up) and report characteristics (e.g., years considered, language, publication status) used as criteria for eligibility, giving rationale. | 5 |
| Information sources | 7 | Describe all information sources (e.g., databases with dates of coverage, contact with study authors to identify additional studies) in the search and date last searched. | 5 |
| Search | 8 | Present full electronic search strategy for at least one database, including any limits used, such that it could be repeated. | 5 |
| Study selection | 9 | State the process for selecting studies (i.e., screening, eligibility, included in systematic review, and, if applicable, included in the meta-analysis). | 5 |
| Data collection process | 10 | Describe method of data extraction from reports (e.g., piloted forms, independently, in duplicate) and any processes for obtaining and confirming data from investigators. | 5 |
| Data items | 11 | List and define all variables for which data were sought (e.g., PICOS, funding sources) and any assumptions and simplifications made. | 5-6 |
| Risk of bias in individual studies | 12 | Describe methods used for assessing risk of bias of individual studies (including specification of whether this was done at the study or outcome level), and how this information is to be used in any data synthesis. | 6 |
| Summary measures | 13 | State the principal summary measures (e.g., risk ratio, difference in means). | 6 |
| Synthesis of results | 14 | Describe the methods of handling data and combining results of studies, if done, including measures of consistency (e.g., I^2^) for each meta-analysis. | 6 |
| Risk of bias across studies | 15 | Specify any assessment of risk of bias that may affect the cumulative evidence (e.g., publication bias, selective reporting within studies). | 6 |
| Additional analyses | 16 | Describe methods of additional analyses (e.g., sensitivity or subgroup analyses, meta-regression), if done, indicating which were pre-specified. | N/A |
| **RESULTS** | | |  |
| Study selection | 17 | Give numbers of studies screened, assessed for eligibility, and included in the review, with reasons for exclusions at each stage, ideally with a flow diagram. | 7 |
| Study characteristics | 18 | For each study, present characteristics for which data were extracted (e.g., study size, PICOS, follow-up period) and provide the citations. | 7 |
| Risk of bias within studies | 19 | Present data on risk of bias of each study and, if available, any outcome level assessment (see item 12). | Table 1 |
| Results of individual studies | 20 | For all outcomes considered (benefits or harms), present, for each study: (a) simple summary data for each intervention group (b) effect estimates and confidence intervals, ideally with a forest plot. | Table 1-6  8-10 |
| Synthesis of results | 21 | Present results of each meta-analysis done, including confidence intervals and measures of consistency. | 8 |
| Risk of bias across studies | 22 | Present results of any assessment of risk of bias across studies (see Item 15). | 8 |
| Additional analysis | 23 | Give results of additional analyses, if done (e.g., sensitivity or subgroup analyses, meta-regression [see Item 16]). | N/A |
| **DISCUSSION** | | |  |
| Summary of evidence | 24 | Summarize the main findings including the strength of evidence for each main outcome; consider their relevance to key groups (e.g., healthcare providers, users, and policy makers). | 11-13 |
| Limitations | 25 | Discuss limitations at study and outcome level (e.g., risk of bias), and at review-level (e.g., incomplete retrieval of identified research, reporting bias). | 13-14 |
| Conclusions | 26 | Provide a general interpretation of the results in the context of other evidence, and implications for future research. | 14 |
| **FUNDING** | | |  |
| Funding | 27 | Describe sources of funding for the systematic review and other support (e.g., supply of data); role of funders for the systematic review. | 15 |
| Note: Moher D, Liberati A, Tetzlaff J, Altman DG, The PRISMA Group (2009). Preferred Reporting Items for Systematic Reviews and Meta-Analyses: The PRISMA Statement. PLoS Med 6(7): e1000097. doi:10.1371/journal.pmed1000097 | | | |

| Appendix 2: Search terms used for final search on 04 June 2020 | | | | |
| --- | --- | --- | --- | --- |
| Searches | Search terms | Medline | Cinahl | PubMed |
| #1 | Cerebrovascular Accident OR CVA OR Stroke | 718,805 | 208,541 | 24,038 |
| #2 | COVID-19 OR CORONAVIRUS OR 2019-NCOV | 39,181 | 6,423 | 20,220 |
| #3 | #1 AND #2 | 404 | 3 | 165 |
| #4 | Limiter ENGLISH, HUMAN | 396 | 3 | 164 |

| Appendix 3: Quality appraisal checklist for case series and case report | | | | | | | | | | | | | | | | | | | | | |
| --- | --- | --- | --- | --- | --- | --- | --- | --- | --- | --- | --- | --- | --- | --- | --- | --- | --- | --- | --- | --- | --- |
| Items | | Mao et al., | Beyrouti et al., | Avula et al., | Zhang et al., | Barios Lopez et al., | Oxley et al., | Tunc et al., | Morassi et al., | Wang et al., | Zayet et al., | Fara et al., | Valderrama et al., | Viguier et al., | Sharafi Razavi et al., | Christian Oliver et al., | Gonzalez-Pinto et al., | Moshayedi et al., | Hughes et al., | Gunasekaran et al., | Goldberg et al., |
| Study objective | | | | | | | | | | | | | | | | | | | | | |
| 1. | Was the hypothesis/aim/objective of the study clearly stated? (Yes/Partial/No) | Yes | Yes | Yes | Partial | Yes | Yes | Yes | Yes | Yes | Yes | Yes | Partial | Partial | Partial | Partial | Partial | Partial | Partial | Partial | Yes |
| Study design | | | | | | | | | | | | | | | | | | | | | |
| 2. | Was the study conducted prospectively? (Yes/Unclear/No) | No | No | No | No | No | No | Unclear | No | No | Unclear | Unclear | No | No | No | No | No | No | No | No | No |
| 3. | Were the cases collected in more than one centre? (Yes/Unclear/No) | Yes | No | No | No | No | No | No | No | Unclear | Unclear | Unclear | No | No | No | No | No | No | No | No | No |
| 4. | Were patients recruited consecutively? (Yes/Unclear/No) | Yes | No | No | No | Yes | No | Unclear | Unclear | Yes | No | No | No | No | No | No | No | No | No | No | No |
| Study population | | | | | | | | | | | | | | | | | | | | | |
| 5. | Were the characteristics of the patients included in the study described? (Yes/Partial/No) | Yes | Yes | Yes | Yes | Yes | Yes | Yes | Yes | Yes | Partial | Partial | Partial | Partial | Partial | Partial | Partial | Partial | Partial | Partial | Partial |
| 6. | Were the eligibility criteria (i.e. inclusion and exclusion criteria) for entry into the study clearly stated? (Yes/Partial/No) | Partial | No | Yes | Partial | Partial | Partial | Partial | Partial | Partial | Partial | Partial | No | No | No | No | No | No | No | No | No |
| 7. | Did patients enter the study at a similar point in the disease? (Yes/Unclear/No) | Unclear | Yes | Unclear | Unclear | Unclear | Unclear | Unclear | Unclear | Unclear | Unclear | Unclear | No | No | No | No | No | No | No | No | No |
| Intervention and co-intervention | | | | | | | | | | | | | | | | | | | | | |
| 8. | Was the intervention of interest clearly described? (Yes/Partial/No) | No | No | No | No | No | No | No | No | No | No | No | No | No | No | No | No | No | No | No | No |
| 9. | Were additional interventions (co-interventions) clearly described?  (Yes/Partial/No) | No | No | No | No | No | No | No | No | No | No | No | No | No | No | No | No | No | No | No | No |
| Outcome measure | | | | | | | | | | | | | | | | | | | | | |
| 10. | Were relevant outcome measures established a priori? (Yes/Partial/No) | Yes | Yes | Yes | Yes | Yes | Yes | Yes | Yes | Yes | Yes | Yes | Yes | Yes | Yes | Yes | Yes | Yes | Yes | Yes | Yes |
| 11. | Were outcome assessors blinded to the intervention that patients received? (Yes/Unclear/No) | Unclear | Unclear | Unclear | Unclear | Unclear | Unclear | Unclear | Unclear | Unclear | Unclear | Unclear | Unclear | Unclear | Unclear | Unclear | Unclear | Unclear | Unclear | Unclear | Unclear |
| 12. | Were the relevant outcomes measured using appropriate objective/subjective methods? (Yes/Partial/No) | Yes | Yes | Yes | Yes | Yes | Yes | Yes | Yes | Yes | Yes | Yes | Yes | Yes | Yes | Yes | Yes | Yes | Yes | Yes | Yes |
| 13. | Were the relevant outcome measures made before and after the intervention? (Yes/Unclear/No) | No | No | No | No | No | No | No | No | No | No | No | No | No | No | No | No | No | No | No | No |
| Statistical analysis | | | | | | | | | | | | | | | | | | | | | |
| 14. | Were the statistical tests used to assess the relevant outcomes appropriate? (Yes/Unclear/No) | Yes | No | No | No | No | No | No | No | No | No | No | No | No | No | No | No | No | No | No | No |
| Results and conclusions | | | | | | | | | | | | | | | | | | | | | |
| 15. | Was follow-up long enough for important events and outcomes to occur? (Yes/Unclear/No) | Unclear | Unclear | Unclear | Unclear | Unclear | Unclear | Unclear | Unclear | Unclear | Unclear | Unclear | Unclear | Unclear | Unclear | Unclear | Unclear | Unclear | Unclear | Unclear | Unclear |
| 16. | Were losses to follow-up reported? (Yes/Unclear/No) | No | No | No | No | No | No | No | No | No | No | No | No | No | No | No | No | No | No | No | No |
| 17. | Did the study provided estimates of random variability in the data analysis of relevant outcomes? (Yes/Partial/No) | Yes | No | No | No | No | No | No | No | No | No | No | No | No | No | No | No | No | No | No | No |
| 18. | Were the adverse events reported? (Yes/Partial/No) | No | No | No | No | No | No | No | No | No | No | No | No | No | No | No | No | No | No | No | No |
| 19. | Were the conclusions of the study supported by results? (Yes/Unclear/No) | Yes | Yes | Yes | Yes | Yes | Yes | Yes | Yes | Yes | Yes | Yes | Yes | Yes | Yes | Yes | Yes | Yes | Yes | Yes | Yes |
| Competing interests and sources of support | | | | | | | | | | | | | | | | | | | | | |
| 20. | Were both competing interests and sources of support for the study reported? (Yes/Partial/No) | Yes | Yes | Yes | No | Yes | No | Yes | Yes | Yes | No | Partial | Yes | Partial | Partial | Partial | Partial | Partial | No | No | No |
| Note: This quality appraisal checklist for case series studies developed by Institute of Health Economics. | | | | | | | | | | | | | | | | | | | | | |

| Appendix 4: Functions of blood test and its normal range | | |
| --- | --- | --- |
| Blood tests | Functions | Normal range |
| Erythrocyte sedimentation rate | a test that indirectly measures the degree of inflammation present in the body. Even though ESR is may not be a diagnostic blood test yet it provide general information about the presence or absence of an inflammatory condition [[1](#_ENREF_1)]. | The normal range for ESR should be range from 0 to 15 mm/hr for men who are younger than 50 years of age; 0 to 20 mm/hr in men aged more than 50 and also for women younger than 50 years of age; and 0 to 30 mm/hr in women aged above 50 [[2](#_ENREF_2)]. |
| C-reactive protein | CRP test does not indicate the cause of inflammation, but studies showed that high level of CRP in blood is associated with an increased risk of heart attack therefore, high CRP level could possible indicate there is inflammation cause by COVID-19 in patients’ blood vessels [[3](#_ENREF_3)]. | The normal range for CRP test result are below 3.0 mg/L [[4](#_ENREF_4)]. |
| Ferritin | A test measures the level of ferritin, the major iron storage protein in body. | Normal ferritin levels range from 12 to 300 mg/L for men and 12 to 150 mg/L for women. An elevated ferritin reading indicates chronic inflammation process is on-going [[5](#_ENREF_5)]. |
| Lactic acid dehydrogenase | It is an indicator for tissue damage caused by viral or bacterial inflammation [[6](#_ENREF_6)]; it is a vital in evaluating a possible stroke. | The normal LDH level range between 140 to 333 IU/L, an elevated LDH levels indicate cell damage[[6](#_ENREF_6)]. |
| D-dimer | D-dimer test is a blood test that rule out the presence of a serious blood clot. | The normal range for D-dimer are between 0.25- 0.5 mg/L[[7](#_ENREF_7)]. |
| Fibrinogen | It aids in the diagnosis of suspected clotting or bleeding disorders caused by fibrinogen abnormalities. | The normal range for (200-400 mg/dL)[[8](#_ENREF_8)] |
| Antiphospholipid antibodies | They are antibodies that increase the risk of blood clots [[9](#_ENREF_9)]. | The reference range findings for absence or none detected of autoantibodies if it was less than 15 immunoglobulin G phospholipid unit or less than 12 immunoglobulin M phospholipid unit, or less than 12 immunoglobulin A phospholipids units [[10](#_ENREF_10)]. |
| Procalcitonin | A test is used for diagnosis of bacterial sepsis. | In healthy people, procalcitonin concentrations are typically below 0.05 ng/mL and an elevated procalcitonin concentrations indicate systemic inflammatory response syndrome and if the value increase up to 1000 ng/mL, the patients could suffer severe sepsis and septic shock [[11](#_ENREF_11)]. |
| Interleukin 6 | A test which useful marker of immune system activation. Elevated reading of interleukin 6 could be due to cardiovascular disease, inflammation, infection and autoimmune disorder. | The normal range of interleukin 6 should be in range of 0 to 16.4 pg/mL. Studies reported that concentration of interleukin 6 is elevated in patients with infection, sepsis and septicaemia [[12](#_ENREF_12)]. |
| Troponin | This test measures the levels of trophonin T or trophonin I protein in the blood. An elevated trophonin value (above 400 pg/mL or 0.4 ng/mL) indicates the heart muscle has been damaged and therefore patients would suffering heart attack, the more damage there is to the heart, the higher concentration of troponin T and I there will be in the blood [[13](#_ENREF_13)]. | Troponin The normal range is below 40 pg/mL or 0.04 ng/mL. |
| Platelet | A platelet count test is used to determine the number of platelets in blood, it is often performed as part of a general health exam and to screen for or diagnose various diseases and conditions that can cause problems with blood clot formation. | The normal range platelet between 150 to 400 x10^9^ [[14](#_ENREF_14)] |
| Prothrombin time | This test helps to detect a bleeding disorder or excessive clotting disorder. | The normal range for clotting is 11 to 13.5 seconds [[15](#_ENREF_15)]. |

**REFERENCES**

1. SOX HC, Liang MH: **Diagnostic decision: the erythrocyte sedimentation rate: guidelines for rational use**. *Annals of internal medicine* 1986, **104**(4):515-523.

2. Brigden ML: **Clinical utility of the erythrocyte sedimentation rate**. *American family physician* 1999, **60**(5):1443-1450.

3. Du Clos TW: **Function of C-reactive protein**. *Annals of medicine* 2000, **32**(4):274-278.

4. Black S, Kushner I, Samols D: **C-reactive protein**. *Journal of Biological Chemistry* 2004, **279**(47):48487-48490.

5. Knovich MA, Storey JA, Coffman LG, Torti SV, Torti FM: **Ferritin for the clinician**. *Blood reviews* 2009, **23**(3):95-104.

6. Drent M, Cobben N, Henderson R, Wouters E, van Dieijen-Visser M: **Usefulness of lactate dehydrogenase and its isoenzymes as indicators of lung damage or inflammation**. *European Respiratory Journal* 1996, **9**(8):1736-1742.

7. Pagana KD, Pagana TJ: **Mosby's Manual of Diagnostic and Laboratory Tests-E-Book**: Elsevier Health Sciences; 2017.

8. Verhovsek M, Moffat KA, Hayward CP: **Laboratory testing for fibrinogen abnormalities**. *American journal of hematology* 2008, **83**(12):928-931.

9. Misita CP, Moll S: **Antiphospholipid antibodies**. *Circulation* 2005, **112**(3):e39-e44.

10. Greaves M: **Antiphospholipid antibodies and thrombosis**. *The lancet* 1999, **353**(9161):1348-1353.

11. Jin M, Khan AI: **Procalcitonin: uses in the clinical laboratory for the diagnosis of sepsis**. *Laboratory Medicine* 2010, **41**(3):173-177.

12. Harbarth S, Holeckova K, Froidevaux C, Pittet D, Ricou B, Grau GE, Vadas L, Pugin J, Network GS: **Diagnostic value of procalcitonin, interleukin-6, and interleukin-8 in critically ill patients admitted with suspected sepsis**. *American journal of respiratory and critical care medicine* 2001, **164**(3):396-402.

13. Wens SC, Schaaf GJ, Michels M, Kruijshaar ME, Van Gestel TJ, in ‘t Groen S, Pijnenburg J, Dekkers DH, Demmers JA, Verdijk LB: **Elevated plasma cardiac troponin T levels caused by skeletal muscle damage in Pompe disease**. *Circulation: cardiovascular genetics* 2016, **9**(1):6-13.

14. Monreal M, Lafoz E, Casals A, Ruíz J, Arias A: **Platelet count and venous thromboembolism: a useful test for suspected pulmonary embolism**. *Chest* 1991, **100**(6):1493-1496.

15. Kamal AH, Tefferi A, Pruthi RK: **How to interpret and pursue an abnormal prothrombin time, activated partial thromboplastin time, and bleeding time in adults**. In: *Mayo Clinic Proceedings: 2007*: Elsevier; 2007: 864-873.
